# Nasal sill augmentation: an overlooked concept in rhinoplasty—a technical note and review of the literatures

**DOI:** 10.1186/s40902-021-00298-3

**Published:** 2021-05-18

**Authors:** Gholamhossein Adham, Seied Omid Keyhan, Hamid Reza Fallahi, Heliya Ziaei, Mohan Thomas

**Affiliations:** 1 Department of Oral and Maxillofacial Surgery, Dental School, Guilan University of Medical Sciences, Rasht, Iran; 2 Maxillofacial Surgery and Implantology and Biomaterial Research Foundation, Tehran/Isfahan, Iran; 3 Dental Research Center, Research Institute of Dental Sciences, Shahid Beheshti University of Medical Sciences, Tehran, Iran; 4 Dental Research Center, Faculty of Dentistry, Tehran University of Medical Sciences, Tehran, Iran; 5 Mount Sinai School of Medicine, New York, USA; 6 The Cosmetic Surgery Institute, Mumbai, India

**Keywords:** Rhinoplasty, Sill graft, Cartilage graft, Nasal sill, Sill augmentation

## Abstract

**Background:**

Nasal sill is one of the components of the alar ring, affecting the esthetic outcomes of rhinoplasty; accordingly, we developed a novel technique to adjust defects in this area and compared it with the available techniques.

**Methods:**

Our technique was based on creating a tunnel access to the nasal sill area through an incision made in the lower third of the columella using the open approach or through a nostril base incision in patients, who underwent alar base reduction, followed by insertion of a cartilaginous graft into the marked defect area.

**Results:**

A total number of 54 patients with a defect in the nasal sill area were included in this study. Thirty-one patients underwent open rhinoplasty with the sill approach from the lower third of the columella, while 23 patients underwent rhinoplasty with a nostril base approach for nasal sill augmentation procedure. There were no reports of patient dissatisfaction, infection, bleeding, sensory dysfunction, or remaining asymmetry of the sill area.

**Conclusion:**

Based on the findings of the present study, this technique can be successfully used in reconstructing the nasal sill area with minimal complications and morbidity.

**Supplementary Information:**

The online version contains supplementary material available at 10.1186/s40902-021-00298-3.

## Background

Esthetic rhinoplasty has become one of the most popular surgeries among candidates for cosmetic surgery [1]. Research on rhinoplasty has increased over the past decades, and surgeons and researchers are paying particular attention to new methods and approaches for achieving the best clinical outcomes. According to our literature review, the majority of published studies have investigated esthetic rhinoplasty and reconstructive surgical procedures [2]. Overall, attention to the anatomy of cartilages and bones in the nasal area, which plays an important role in the esthetics and/or function of the nose, can positively affect the outcomes of the rhinoplasty.

A comprehensive understanding of the unique anatomy of nasal sills is very important, as it exerts prominent effects on the size and shape of nostrils, and more generally, on the esthetic outcomes of nasal procedures [3, 4]. So far, several techniques have been introduced for nasal sill reduction. Nasal sill excision, with or without alar wedge excision, is a method for decreasing the amount of alar flare and also changing the nostril size, shape, and angle as part of the rhinoplasty procedure [5]. However, care must be provided during these procedures, as improper resection of the nasal sill soft tissue can result in deformities, such as tear-shaped nostrils [6].

On the contrary, nasal sill area augmentation has not received much attention so far, although the use of this technique is quite practical for cases, such as uni/bilateral cleft lip or cleft lip and palate, traumatic lesions, congenital asymmetry of the nostril size, secondary revision surgeries, and reconstruction of defects caused by malignancies (e.g., basal cell carcinoma) [7]. So far, few methods and techniques have been introduced for the nasal sill graft procedure [5, 8–10]. Therefore, in this study, we aimed to introduce our novel sill graft technique that can be safely used for most of the aforementioned cases.

This study aims to introduce a novel technique for nasal sill augmentation and compare it with previous techniques briefly. The hypothesis is that the clinical outcome of this technique is optimal esthetic results with minimal morbidity and postoperative complications.

## Methods

### Indications

1. During esthetic rhinoplasty, the need for nasal sill grafting/augmentation may be considered by a surgeon during an esthetic surgical procedure on the nose. For instance, when esthetic rhinoplasty is undertaken to reduce the width of the columellar base, the soft tissue complex, including muscles, ligaments, fibrosis tissue, and fat, is removed. The surgeon then reduces the width of the columellar base by suturing the structures in close proximity. The possibility of hollowing in the sill area can be one of the side effects of this process that must be considered by surgeons, and appropriate measures must be taken to adjust it. Overall, the sill graft can be considered as an appropriate treatment option for this condition.
2. In patients with cleft lip and palate, the lack of a well-defined nostril sill is often due to the presence of a poor-quality scar tissue. The decision about the type of graft is made, depending on the quality of soft tissue in the area. Scarred and adherent soft tissue quality may require the use of a chondrocutaneous composite graft for the reconstruction procedure.
3. Severe deviation of the nasal septum is another factor that may significantly affect the bulging of the sill area. One of the most serious complications of this condition is the asymmetry of the nostril sills, which may not be even compensated by the correction of the nasal septum deviation and may require separate reconstruction.
4. Inherent or trauma-related nasal sill deformities, besides deformities due to malignancies.

### Surgical technique

All procedures were performed by an oral and maxillofacial surgeon in the hospital setting during 2018–2020. Informed consent was obtained after the surgical procedure, potential risks, and complications were described to all patients. The surgery was performed under general anesthesia. Local anesthesia (2% lidocaine with epinephrine at a concentration of 1:80,000) was administered in the surgical field.

**Fig. 1:**
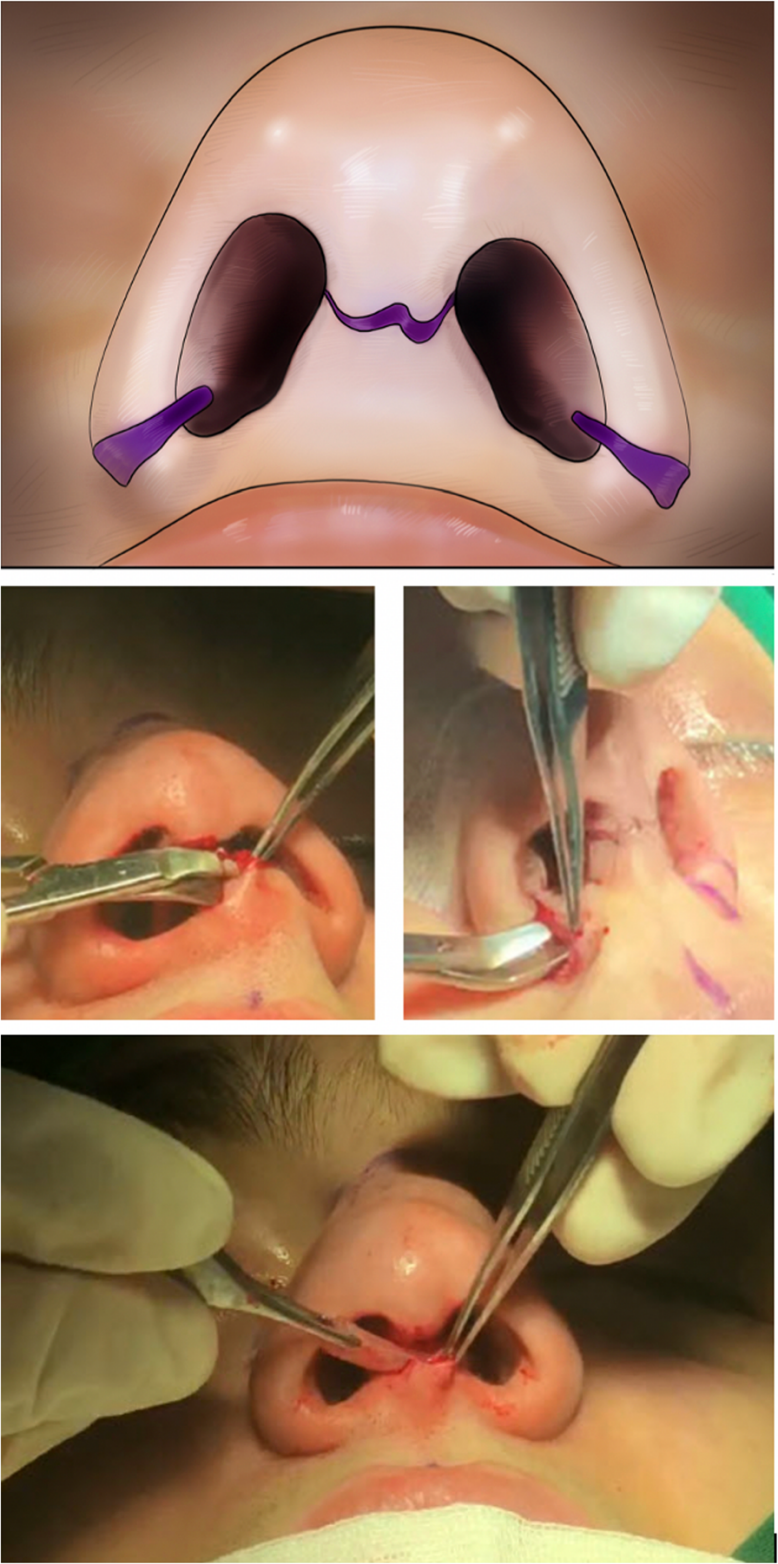
**a** Approaches to access the sill area. **b** Access to the sill area through the incision made in the lower third of the columella in the open rhinoplasty approach. **c** Access to the sill area from the incision at the nostril base in a patient who underwent alar base reduction. **d** Hemostasis is achieved, and an adequately shaped cartilage graft harvested from the septal cartilage or the conchal cartilage is inserted into the tissue pocket. Septal cartilage is considered as the most common source for the harvesting of cartilage graft in rhinoplastic procedures

The cartilaginous graft is harvested from the nasal septum in the priority for each patient. If it is not possible, cartilaginous ear grafts, allografts, and even autogenous rib grafts were the next donor sites consecutively. In the open approach, access to the sill area was achieved through a rhinoplasty incision made, in the lower third of the columella. Also, access to the sill area in patients, who underwent alar base reduction, was achieved through an incision in the nostril base area (Fig. 1). A subcutaneous pocket was dissected from the deficient nasal sill, using converse dissecting scissors.

Commonly, due to the presence of scar tissue and inadequate normal soft tissue in cleft lip and palate patients, there is often a need for scar revision and muscle repositioning. However, since this study aimed to show that nasal sill augmentation is not necessarily limited to cleft patients, individuals with simple congenital asymmetry in the sill area were also selected for photographic evaluation.

The nasal sill augmentation process is not usually performed as an independent procedure and is often a complementary corrective procedure during rhinoplasty or cleft lip repair process, after ensuring the proper form and symmetry of the ala. The remaining steps of the procedure, such as suturing incisions and dressing, are performed during standard rhinoplasty procedures.

For more information about different steps and variations of this technique, the supplemental materials section of this article is available on the website of the Journal of Oral and Maxillofacial Surgery (videos 1 and 2).


**Additional file 1: Video 1** Nasal sill augmentation through nostril base approach.


**Additional file 2: Video 2** Nasal sill augmentation through columellar approach.

### Literature review

A literature review was performed to evaluate previously described techniques addressing for nasal sill deficiency. A comprehensive search was undertaken in PubMed and Scopus databases, without any time restrictions, using keywords related to reconstruction, augmentations, or graft procedures in the nasal sill area, which are as follows: (“nostril sill” OR “nasal sill”) AND (“Reconstructive Surgical Procedures” OR augmentation OR reconstruction OR graft).

**Fig. 2:**
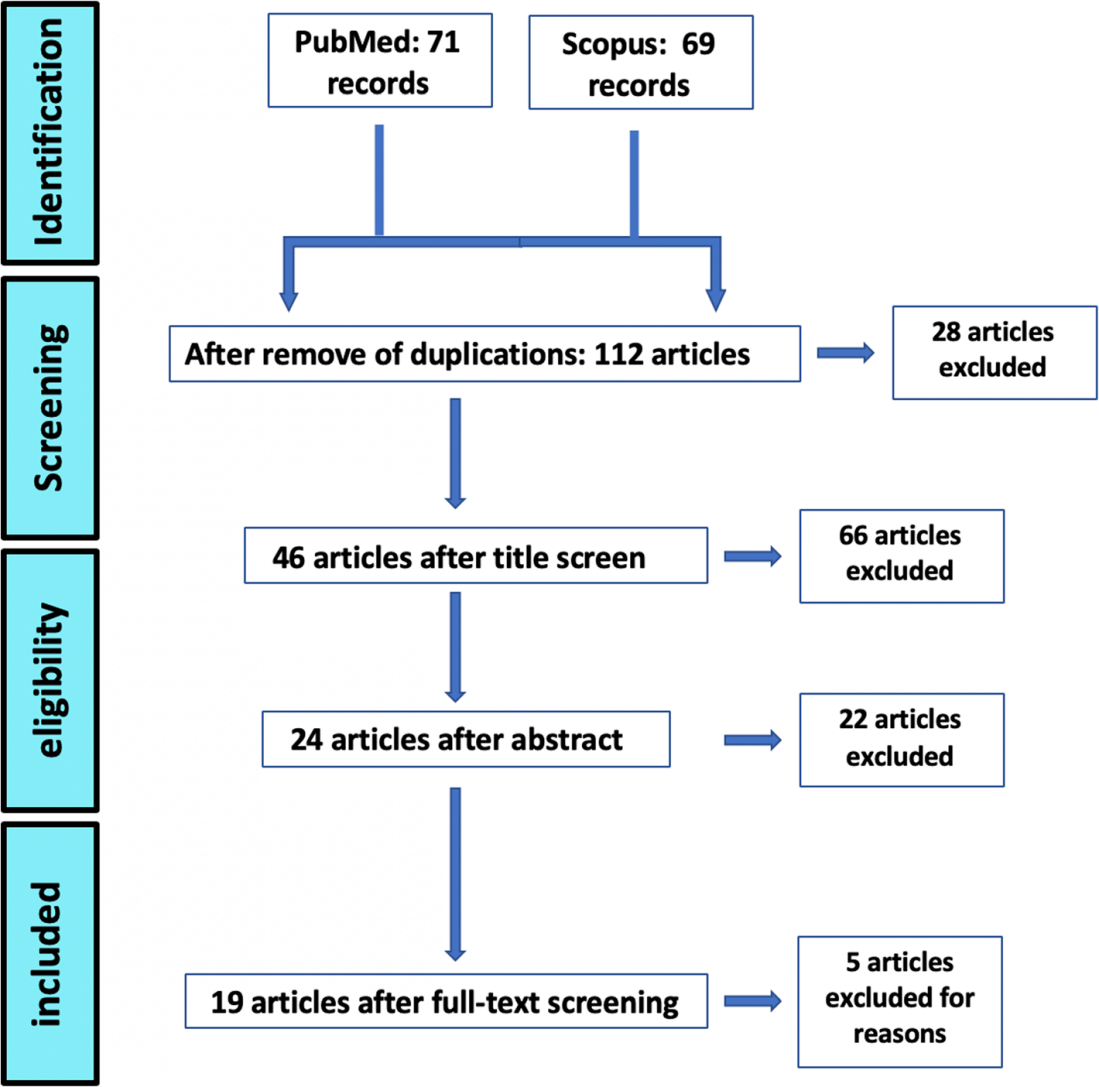
PRISMA flow chart

Figure 2 presents the PRISMA flowchart regarding the search results and screening. Title, abstract, and full-text screening was performed by two independent researchers. Finally, a total of 19 articles were included in this study.

## Results

A total number of 54 patients (19 males and 35 females) with a defect in the nasal sill area were included in this study. The mean age of the patients was 31 years. The candidates for nasal sill augmentation included esthetic rhinoplasty patients (*n*=21), patients with cleft lip nasal deformities (*n*=12), patients with congenital sill defects (asymmetry or absence of the sill) (*n*=9), patients who underwent removal of a malignant lesion (*n*=5), and patients with traumatic injuries (*n*=7). Thirty-one patients underwent open rhinoplasty; therefore, access to the sill graft was achieved from the lower third of the columella. Also, 23 patients underwent the nasal sill augmentation procedure, using a nostril base approach.

**Fig. 3:**
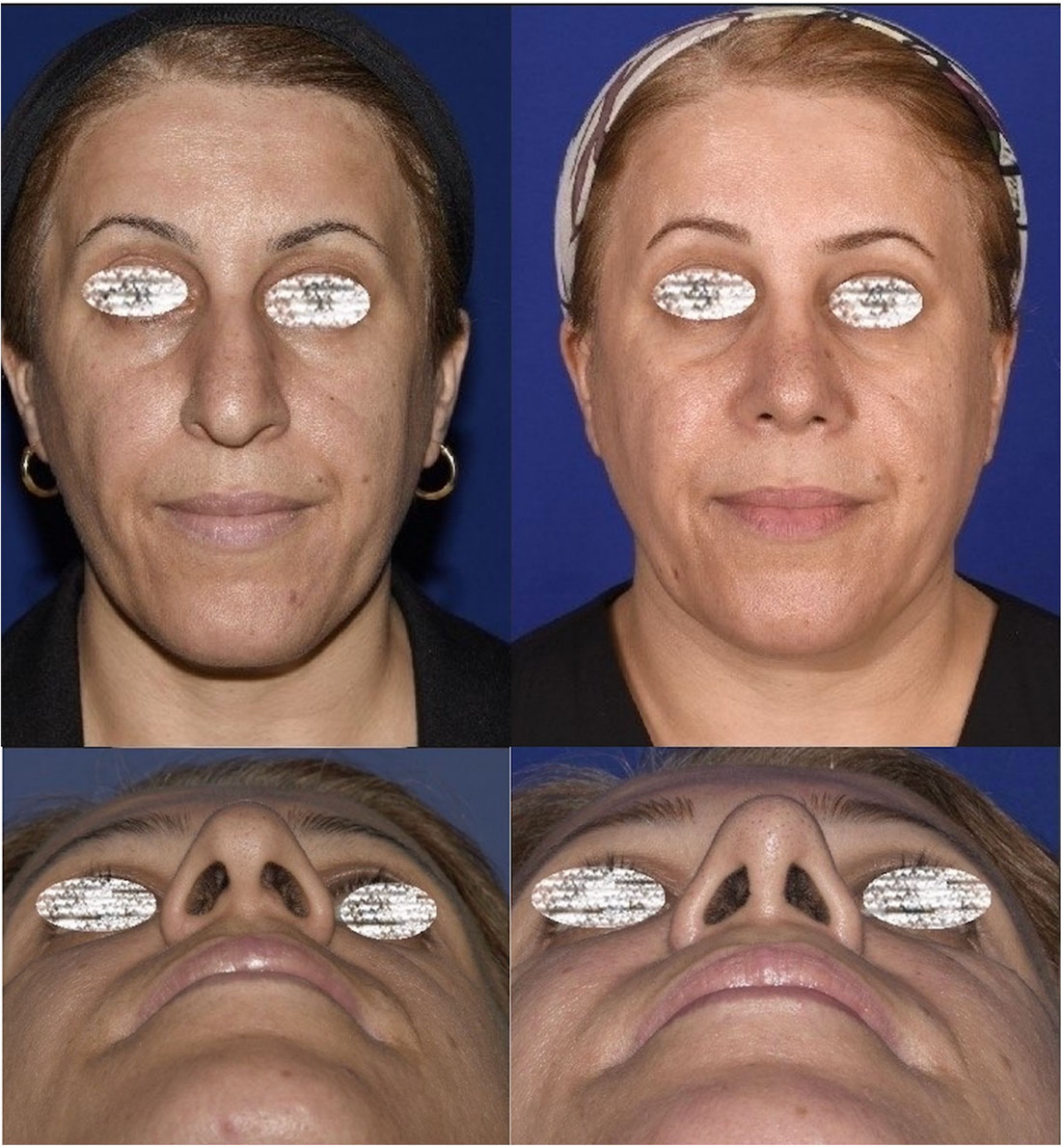
Patient with congenial asymmetry of the nasal sill area who underwent sill graft procedure. The figure shows preoperative and postoperative views from basal aspect (**a**, **b**) and frontal aspect (**c**, **d**). **b**, **d** Postoperative views

**Fig. 4:**
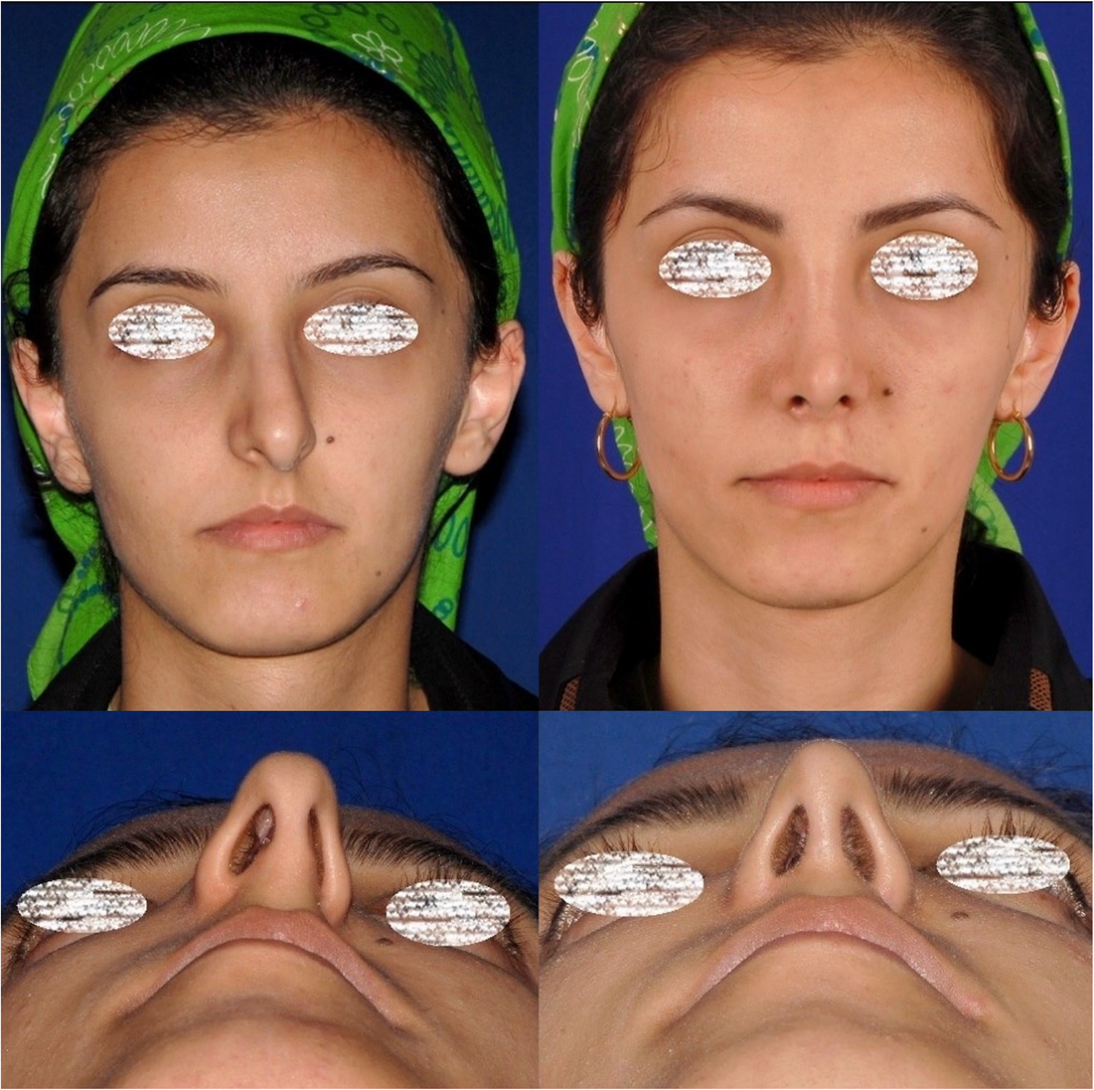
Patient who underwent rhinoplasty with sill graft in preoperative and postoperative views from frontal (**a**, **b**) and basal (**c**, **d**) aspects. **b**, **d** Postoperative views

**Fig. 5:**
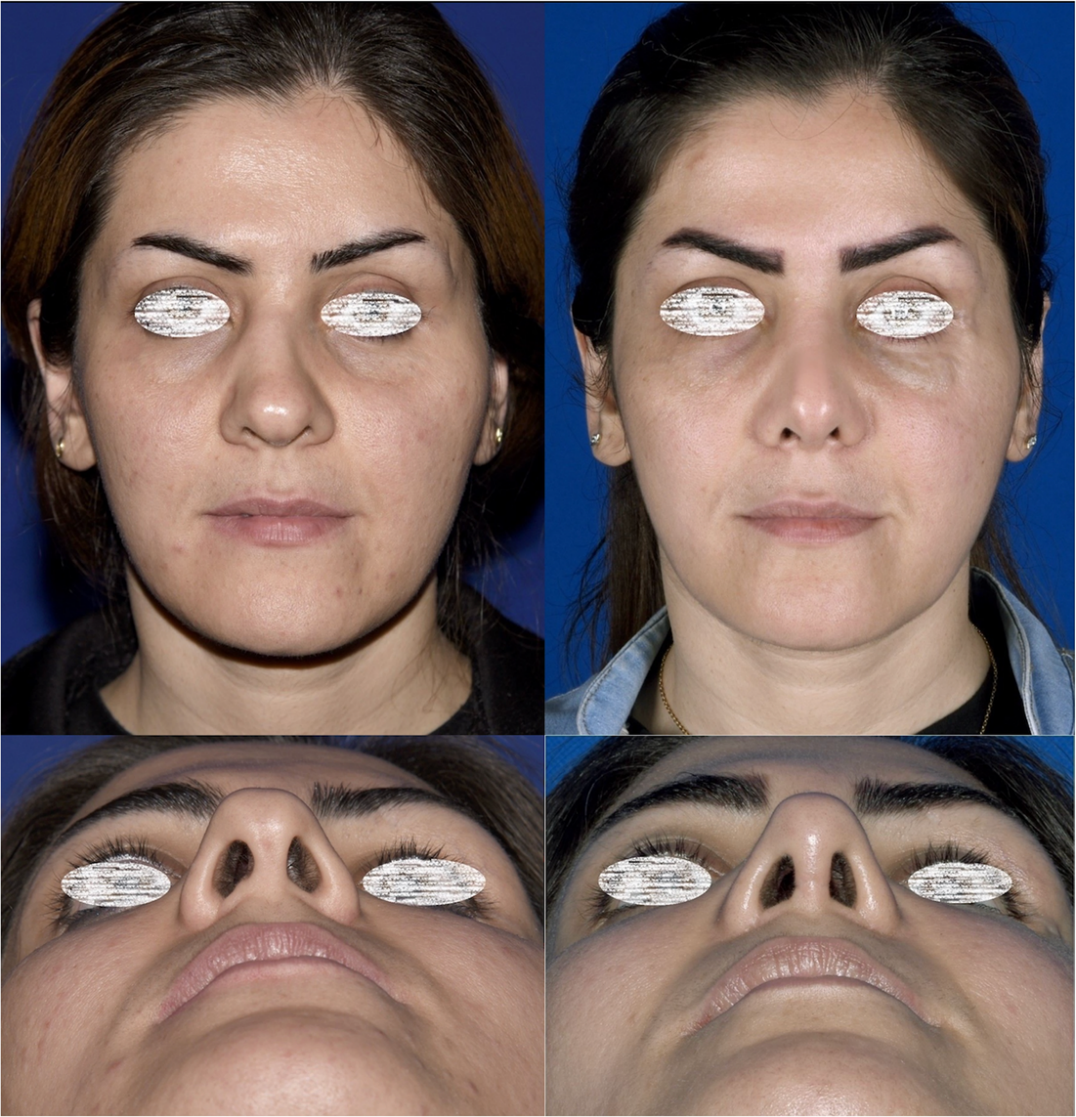
Patient with congenial asymmetry of the nasal sill area who underwent sill graft with alar base approach. The figure shows preoperative and postoperative views from frontal aspect (**a**, **b**) and basal aspect (**c**, **d**). **b**, **d** Postoperative views

**Fig. 6:**
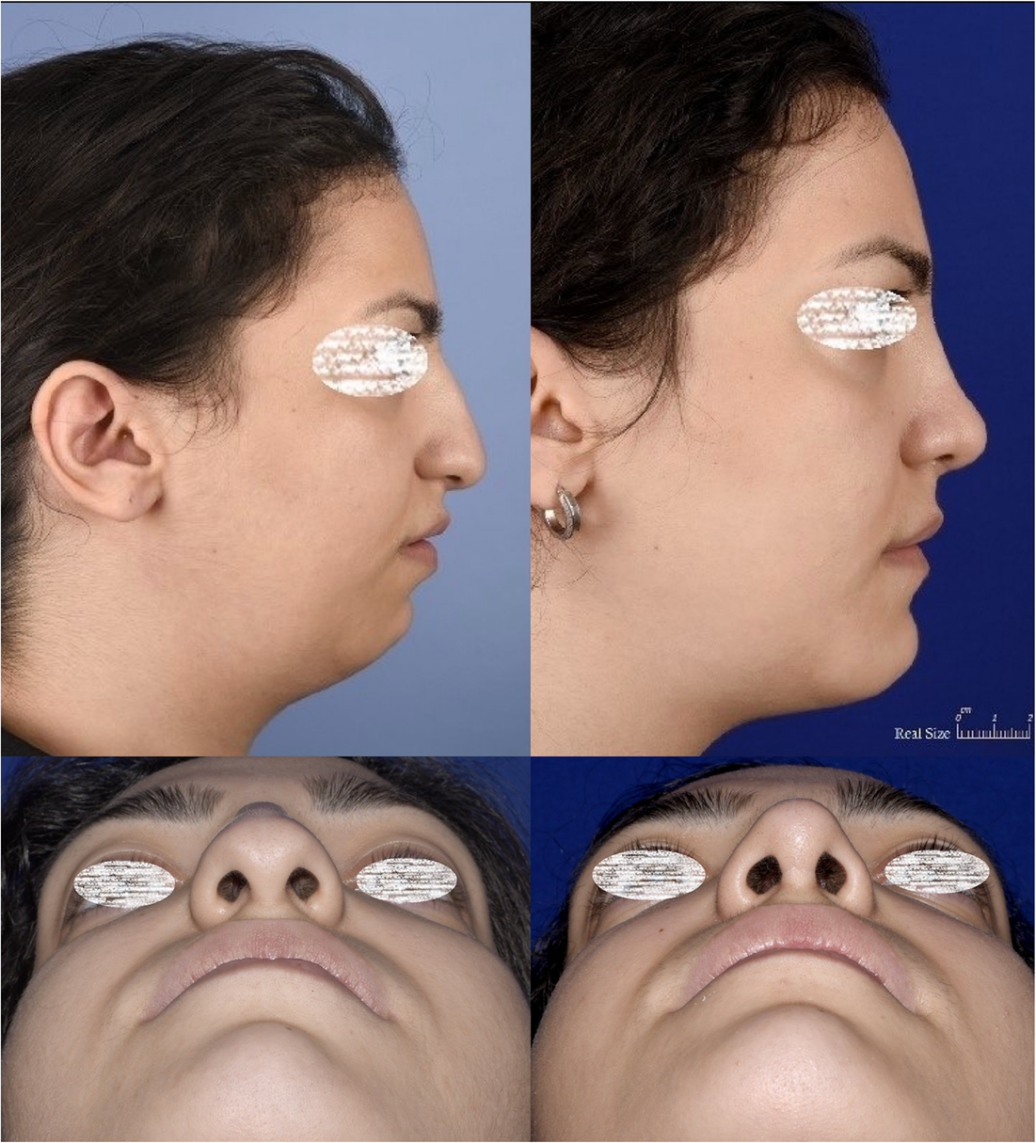
Patient who underwent rhinoplasty with sill graft. The figure shows preoperative and postoperative views from frontal aspect (**a**, **b**) and basal aspect (**c**, **d**). **b**, **d** Postoperative views

**Fig. 7:**
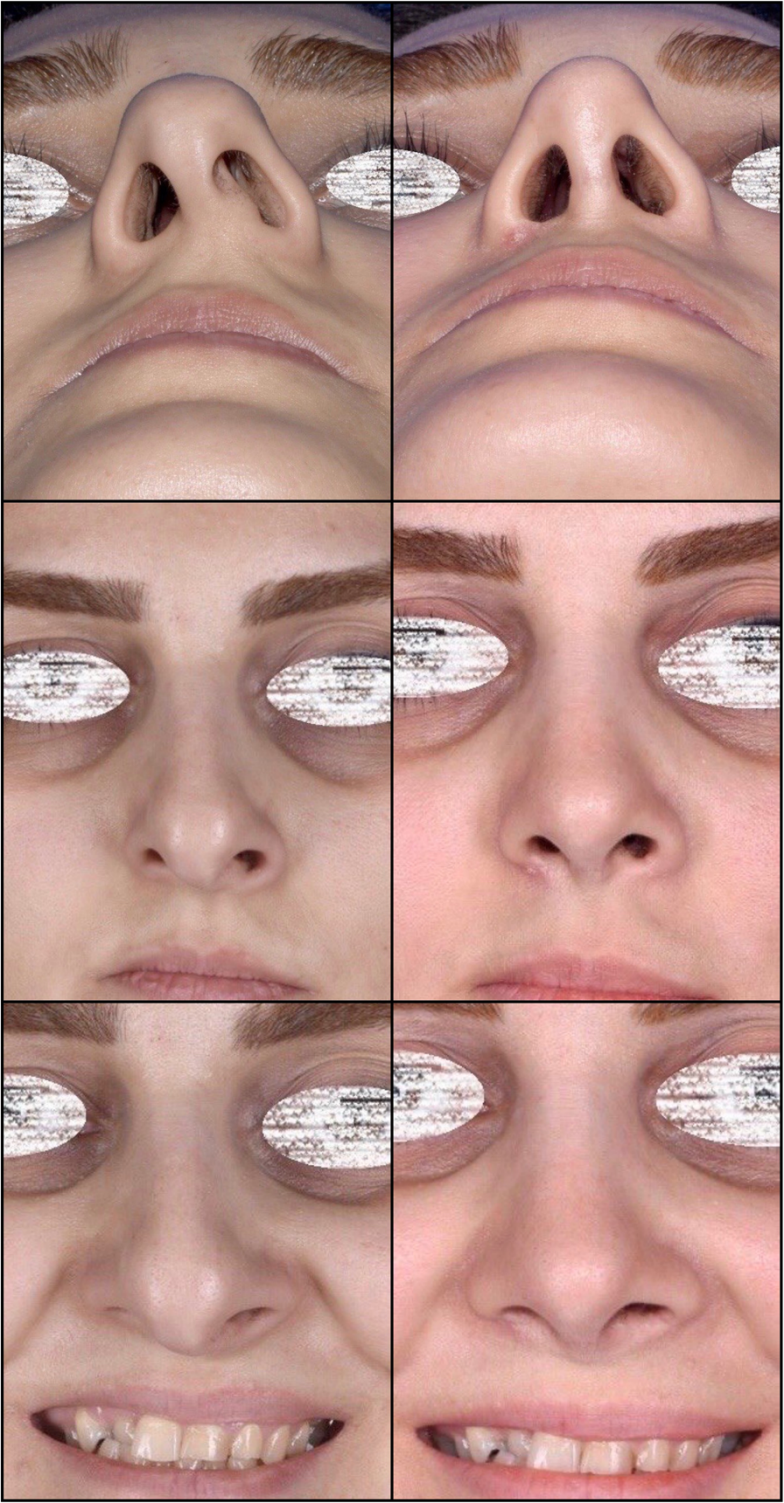
Patient who underwent rhinoplasty with sill graft. The figure shows preoperative and postoperative views from lateral aspect (**a**, **b**) and basal aspect (**c**, **d**). **b**, **d** Postoperative views

The follow-up duration ranged from 11 months to 3 years (mean 1.3 years). The same surgeon evaluated the patients in the follow-up sessions. The patients’ satisfaction was evaluated using the Rhinoplasty Outcome Evaluation Form (ROF) by Izu et al., adapted from a study by Guillemin et al. [11, 12]. Table 1 presents the mean scores of patients’ satisfaction at the end of the follow-up. There was no report of patients’ dissatisfaction at the end of the follow-up. Also, there were no significant complications, such as infection, bleeding, sensory dysfunction, or remaining asymmetry of the sill area. Figures 3, 4, 5, 6, and 7 present the final outcomes at the end of the follow-up in five patients, who underwent sill graft procedures. Table 2 presents the findings of articles, addressing the sill graft/augmentation in terms of the techniques, approaches, clinical results, and complications.

**Table 1:** The mean score of patients’ satisfactions who filled the Rhinoplasty Evaluation Form (ROF) at the end of their follow-up period

Questions	Answers	Mean Score
Question 1: Do you like how your nose looks?	Absolutely no (0), A little (1), More or less (2), Very much (3), Absolutely yes (4)	3.2 ± 0.4
Question 2: Do you breathe well through your nose?	Absolutely no (0), A little (1), More or less (2), Very much (3), Absolutely yes (4)	3.5 ± 0.1
Question 3: Do you believe your friends and people who are dear to you like your nose?	Absolutely no (0), A little (1), More or less (2), Very much (3), Absolutely yes (4)	2.9 ± 0.8
Question 4: Do you think the current appearance of your nose hampers your social or professional activities?	Always (0), Frequently (1), Sometimes (2), Rarely (3), Never (4)	3.1 ± 0.3
Question 5: Do you think your nose looks as good as it could be?	Absolutely no (0), A little (1), More or less (2), Very much (3), Absolutely yes (4)	3.3 ± 0.4
Question 6: Would you undergo surgery to change the appearance of your nose or to improve your breathing?	Certainly yes (0), Very likely yes (1), Possibly yes (2), Probably no (3), Certainly no (4)	3.6 ± 0.2

**Table 2:** The data of articles addressing the sill graft/augmentation in terms of the technique, approaches, clinical results, and complications. *UCL*, unilateral cleft lip; *BCC*, basal cell carcinoma; *AB*, alar base; *NCR*, no complications reported

Deformity	Technique	***N***	Approach	Follow-up	Clinical outcomes	Complications	Ref.
UCL	Flap repositioning following dissecting depressor septi and the medial orbicularis oris muscles	378	AB (along the scar line)	12 months	Stable and natural form of the nostrils and nasal sill	NCR	[13]
UCL	Use of Millard method for correction of the upper part of the lip Elevation of superiorly based scar flap Creation of soft tissue pocket in the nostril floor Folding and insertion of scar flap into the pocket Flap securing with a pull-out stich	16	AB (along the scar line)	Up to 4 years	Acceptable esthetic outcomes	Long lip Drooping of the cleft side Pyriform gap	[3]
UCL	Double Composite Tissue Z-plasty using ilium, rib or costicartilage graft if necessary	68	AB (along the scar line)	14.6 months	Symmetric width of the nostrils and nasal sill and correction of septum and columella deviation	2 graft deviation, 4 impaired ventilation, 1 decreased nostril size No complications such as bleeding, infection, flap necrosis, and sensory dysfunction	[10]
UCL	Triangular flap with pedicle on the nasal base transferred medially to reconstruct the nostril sill	9	AB (along the scar line)	No follow-up was reported	Elevation of the sill area was reported, satisfactory results by the patients.	NCR	[14]
UCL	Elevation and subsequent overlapping of medial and lateral orbicularis oris muscle flaps through an intraoral incision without using filling materials; a tight, large-bite suturing of the muscle in the alar base to correct the sill depression	60	AB (along the scar line)	20 months (Mean)	Effective nostril sill Augmenting without graft, minimal scarring	NCR	[15]
UCL	Upper triangular flap Reestablishing of the sillo–columellar distance muscle layers approximation	250	AB (along the scar line)	2 years for 40% of patients	Straight philtral column scar parallel to the noncleft side to hide the surgical scars on the medial aspect of the nostril and in the lip-columellar crease	NCR	[16]
UCL	Muscle Tension Line Group Theory (first and second axillary tension line group) by operation in medial and lateral orbicularis oris muscle.	263	AB (along the scar line)	18 months for 212 patients	In nearly half of the patient’s nasal sill was similar to normal anatomical sill, 17 patients do not have any improvement in the nasal sill appearance.	NCR	[17]
Nose deformity in Incomplete cleft lip	Superiorly based orbicularis oris muscle flap from the soft tissue between the apex of the cleft and the nostril sill anchored to the anterior nasal spine.	18	AB (along the scar line)	18–32 months	Acceptable esthetic result	NCR	[18]
Unilateral cleft lip and palate	Alveolar bone grafting with iliac bone with 2.39 cm^2^ volume with a tension free suture in the flaps.	18	AB (along the scar line)	16.6 months	Nostril sill elevated significantly on both basal and lateral views.	No major complications (infection, graft failure, wound dehiscence or persistence of an oronasal fistula)	[19]
UCL, trauma, malignancies, assymetries	Composite earlobe grafts with sandwiched cartilage grafts, adjuvant hyperbaric oxygen therapy	5	AB (along the scar line)	Not mentioned	Restored nostril symmetry Increased size of nostril opening Improved appearance of deformed nasal ala	Partial epidermolysis of the graft (*N*=1) Patient dissatisfaction (*N*=1)	[20]
Secondary cleft lip nasal deformity	Composite chondrocutaneous grafts; composite auricular conchal cartilage graft was harvested in an elliptical shape with 1 * 1 cm of skin island and 2 × 2.5 cm of cartilage in the base of the graft.	12	Along the scar line in cleft patient Through the involvement area in BCC patients Classic rhinoplasty approach for other patients	6 months to 2 years	Satisfaction of patients in all cases, minimal morbidity in donor site, not specific data about the result of the sill area	Composite graft Protrusion (*N*=1).	[21]
	Primary correction of nasal deformity in unilateral incomplete cleft lip, A comparative study between three techniques	21	Closed rhinoplasty Cartilage dissection and repositioning through lip incisions	5 years	Improvement in the nasal sill area was greater in group 2 and 3 compared to group 1; But the difference is not significant.	NCR	[22]
Incomplete UCL	Primary correction of nasal deformity in unilateral incomplete cleft lip, A comparative study between three techniques Performing 2 mucosal flaps in the upper lip margin, one flap pedicled around the alveolar cleft was horizontally rotated by 90° to approximate its mucosal surface to the oral side. The downside of another mucosal flap was sutured to the mucosal surface of flap D near the labiogingival groove, Orbicularis oris muscle was repositioned	25	A semi-open rhinoplasty technique Cartilage dissection through bilateral rim incisions	5 years 3–6 months	Improvement in the nasal sill area was greater in group 2 and 3 compared to group 1, but the difference is not significant. Full nasal sills in all cases with patients’ satisfaction	NCR Shorter lip height on the cleft side with symmetrical lip length (*N*=4). Patient dissatisfaction about obvious scars on upper lips (*N*=3).	[22, 23]
		20	A semi-open rhinoplasty technique Tajima incision on the cleft side and a Rim incision on the contralateral side				
Complete UCL		45	AB (along the scar line)				
Complete UCL	Straight-Line Advanced Release Technique (StART)	72	AB (along the scar line)	5 years	Symmetry between the sill areas, minimal scar in all cases	NCR	[9]
Unilateral or bilateral complete cleft	Performing 2 medial and lateral upper lip mucosal flaps. The medial flap was sutured to the lateral nasal mucosa, forming the upper layer of the nasal floor. The lateral flap sutured to the tissue cuff of the gingivopalatal mucosa on the greater alveolar segment to form the lower layer of the nasal floor. The orbicularis oris muscle is repositioning. Nostril floor and nasal sill are formed by approximating the alar base flap and the septal flap.	6	AB (along the scar line)	1 year	Symmetry of nostril shape and the fullness of the nostril sill	NCR	[24]
BCC	Nasocheek flap and a septal cartilage graft Additional surgery for reconstructing the sill area after 3 months	1	Through the involvement area	Unknown duration	Columella with excellent contour, reconstructed sill area	No ischemia or congestion No donor site morbidity	[25]
BCC	V-Y advancement flap Inferiorly based tunneled mesiolabial flap	1	Through the involvement area	8 months	Preservation of the alar apical triangle Single-stage procedure Minimized eclabion formation	Central lip elevation	[26]
BCC	A composite alar graft from the intact alar rim was placed in the opposite involved alar rim and a submental full-thickness skin graft was placed in the philtral area and nostril sill	1	Through the involvement area	7 months	Good healing and reconstruction of the alar rim and philtrum, but not significant description regarding the sill area	NCR	[27]
Binder's syndrome	Cartilage graft on the nostril sill area, dorsum, and around the pyriform aperture	2	Intraoral buccal sulcus incision between canines	12 months	Improved nasal profile without scarring the columella	NCR	[28]

## Discussion

The nasal sill area is a key component of the alar ring, which needs to be considered by surgeons during the nasal base reconstruction procedure [13]. Augmentation of this area is indicated when conditions, such as congenital asymmetrical nostrils, cleft lip and palate, malignancies, or traumatic lesions, occur [20].

### Surgical anatomy

**Fig. 8:**
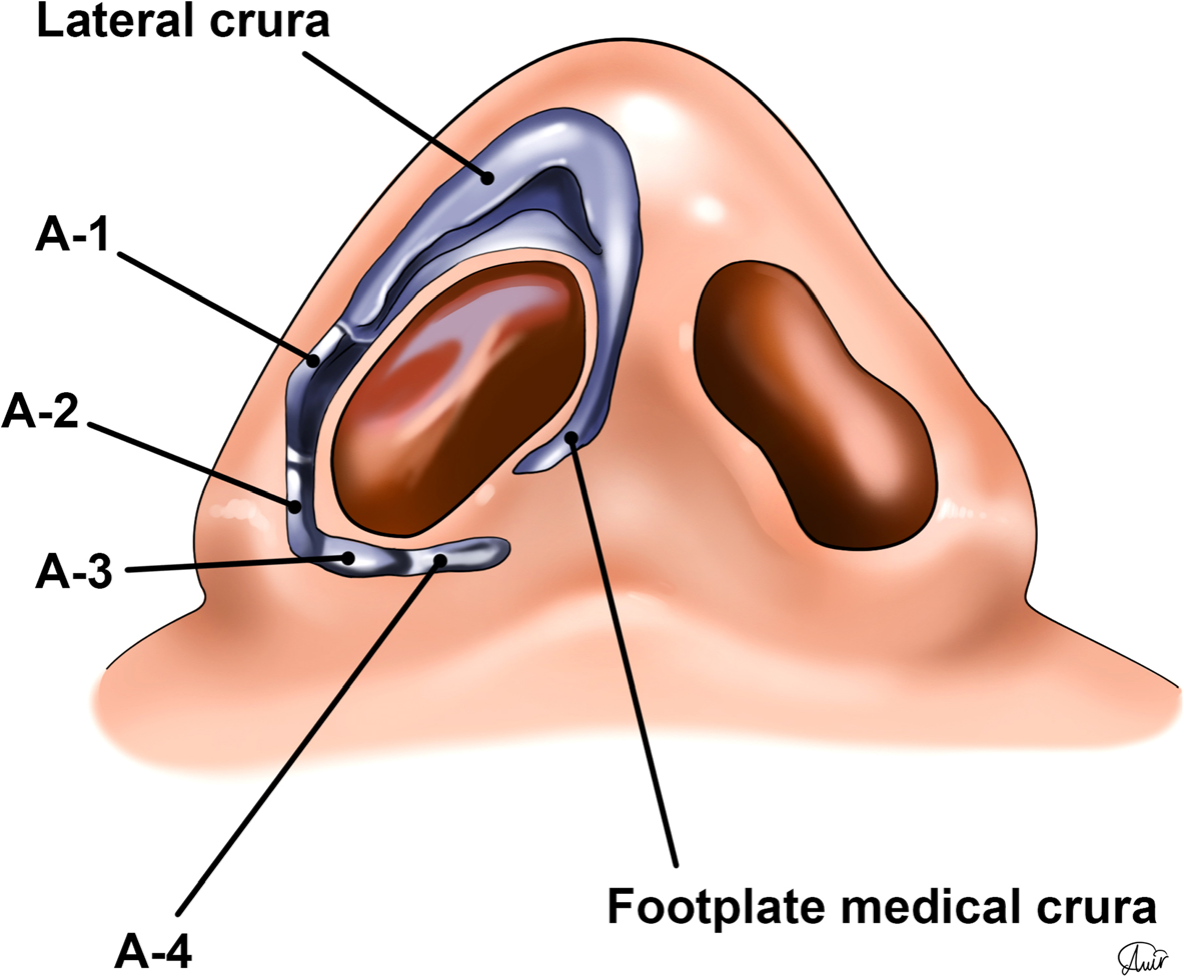
The lateral and medial crura and alar ring and A1 to A4 cartilages, featuring basilar aspect

**Fig. 9:**
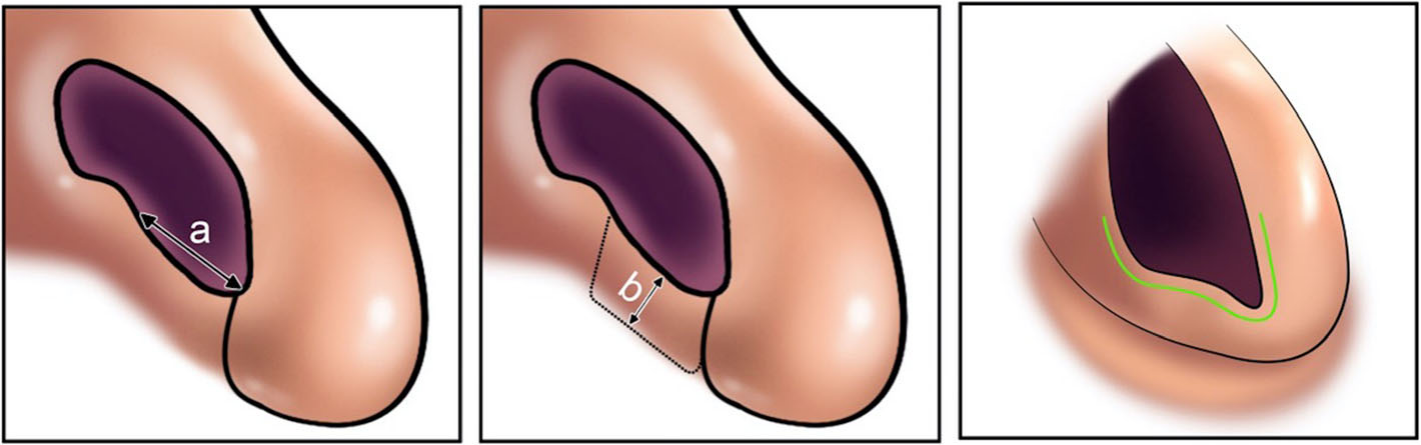
The nasal sill can have variety in terms of (**a**) width (**b**) height, and (**c**) shape

The emphasis on the precise anatomical considerations in the sill area helps the surgeon to have a broader horizon to reach the optimal esthetic results. The alar ring is the most caudal area of the nose, which involves the edge of the nostril, extending to the alar base. It contains the alar cartilage with lateral and medial crura, as well as A1 to A4 accessory cartilages, positioned along the tail of the lateral crural cartilage [29] (Fig. 8). The boundaries of the nostril opening (alar ring) contains alar lobules, columellar base, and nostril sill [30]. As an alar ring subunit, the nasal sill is a protuberant soft tissue bridge, extending from the base of the columella to the ala of the nose, separating the upper lip soft tissue from the nasal vestibule cephalocaudally [30]. The nostril sill is situated approximately in the area of A3 and A4 cartilages. Also, the nostril sill can vary based in terms of width, height, and shape (Fig. 9).

**Fig. 10:**
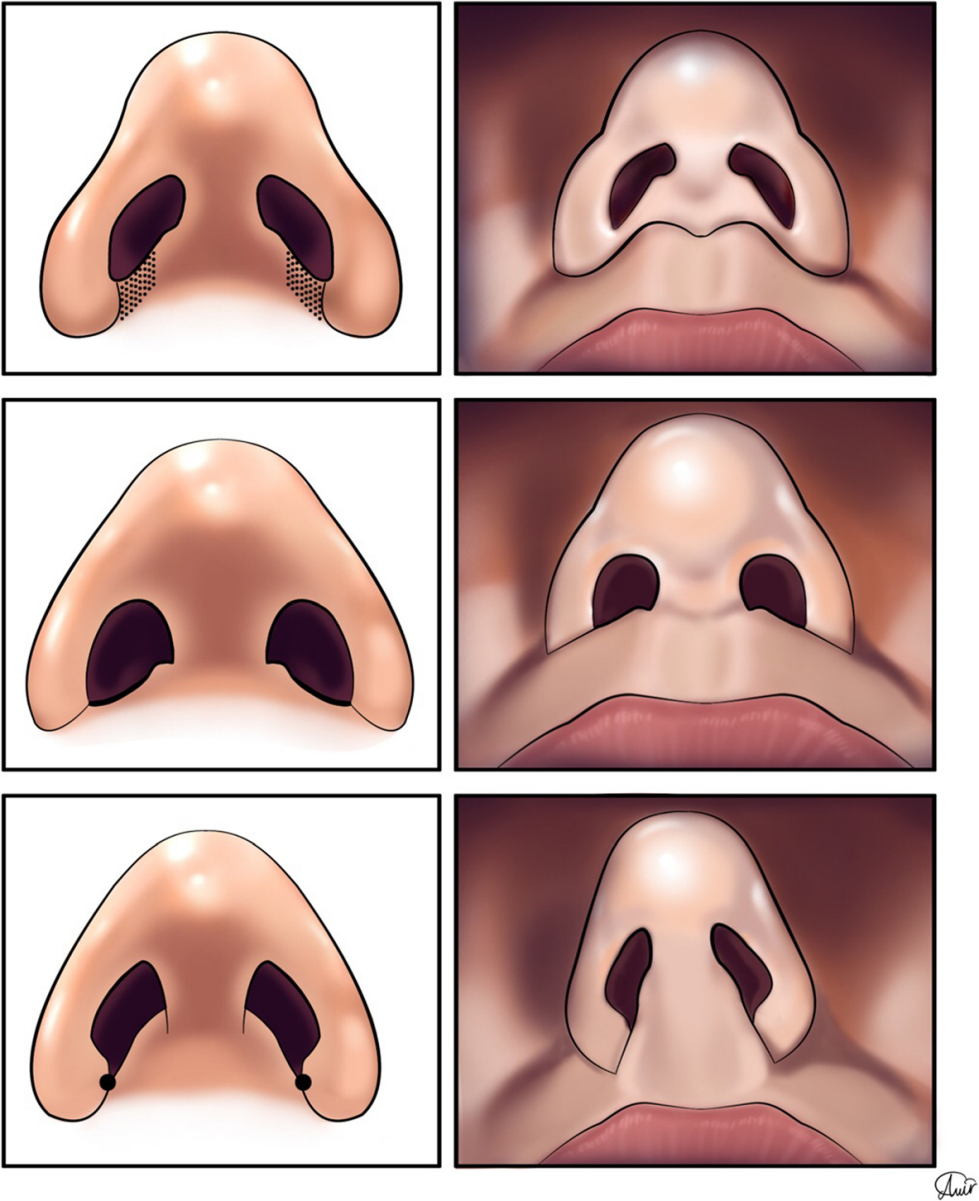
**a** Full or Sill-proper nostril sill which is the most common variant and has the most muscle and soft tissue thickness among all 3 types; a mild protuberant area connects the columella and the ala. **b** In the Flat type, there is no protuberance between the vestibule of the nose and the upper lip and has the least thickness of the soft tissue. **c** In the Point type, the medial and lateral wall of the nostril sill gets close to each other to form an apex

In 1995, Irwin et al. categorized the nostril sill into three main types;

1. In Full/Sill-proper type, a protuberant area connects the columella and the ala. It is the most common variant with the greatest muscle and soft tissue thickness of all three [31].
2. In the Point type, the medial and lateral walls of the nostril sill approximate each other to form an apex.
3. In the Flat type, there is no soft tissue protuberance between the vestibule of the nose and the upper lip, with the least soft tissue thickness [31, 32] (Fig. 10).

**Fig. 11:**
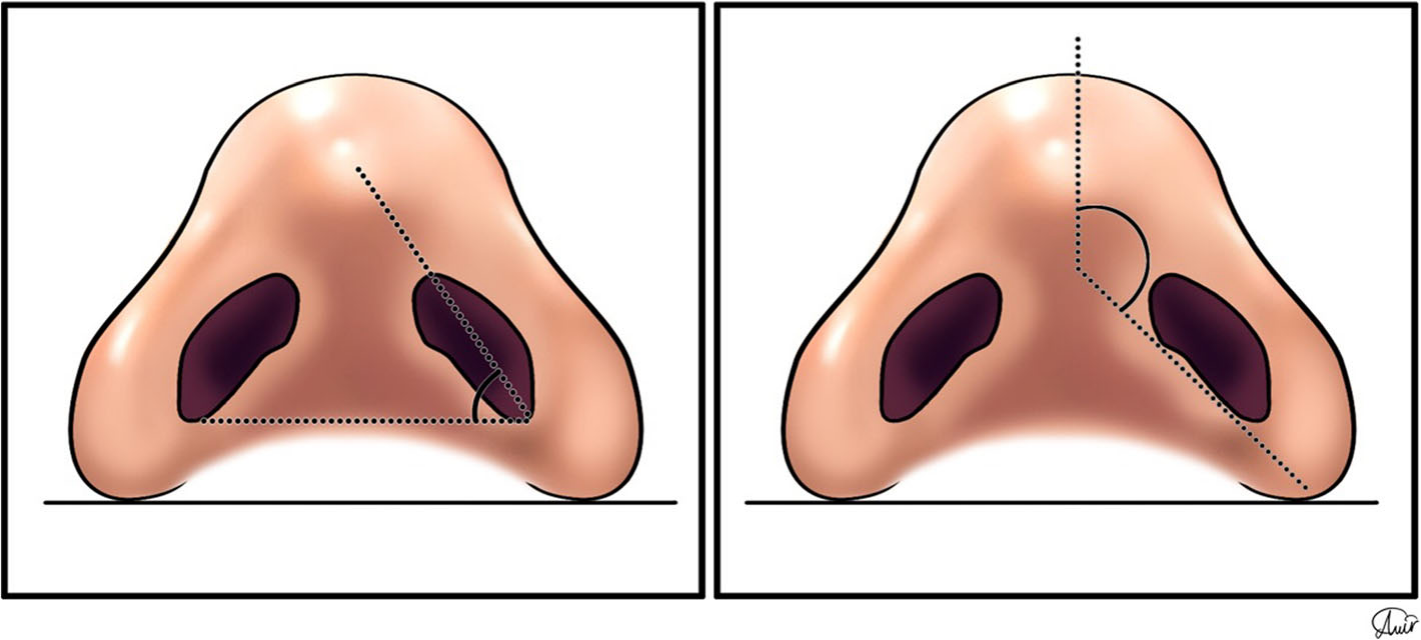
**a** The angle between the longitudinal nostril axis and the horizontal plane and **b** the angle between the line along with the medial inclination of the nasal sill and the sagittal plane. These angles can be considered and recorded in the patient’s preoperative analysis

The direct relationship between the nostril shape and sill area can be inferred from two measurable angles in this area. Figure 11 shows the angle between the longitudinal axis of the nostril and the horizontal plane, and the second angle is between the medially inclined nasal sill and the sagittal plane. An elliptical or pear-shaped nostril with a longitudinal axis angle of 45° has higher esthetic values [6]. These angles can be considered and recorded in the patient’s preoperative analysis.

**Fig. 12:**
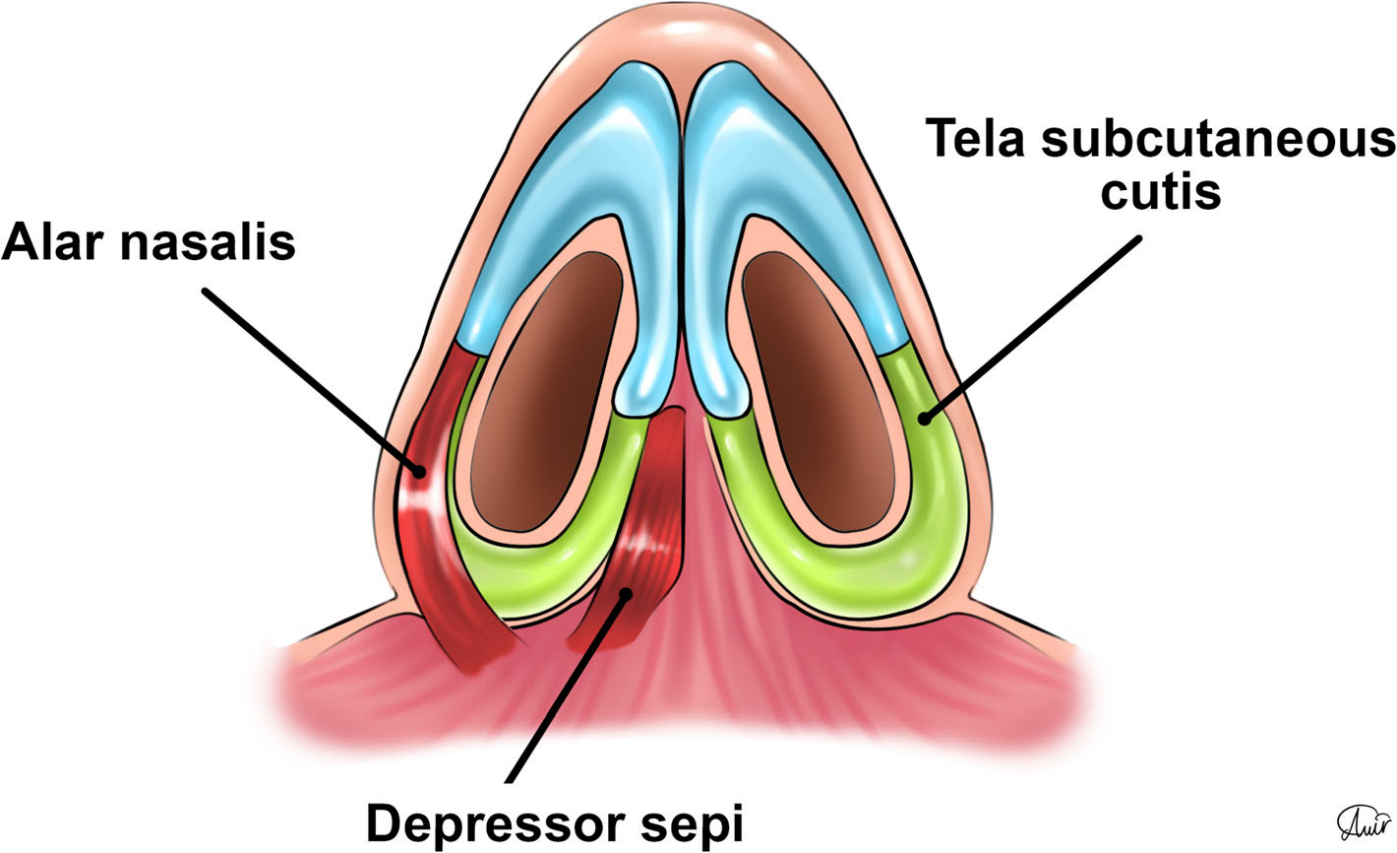
Tela subcutanea cutis, depressor septi nasalis muscles can be seen from basal view; mytriformis and dilator naris cannot be seen from this view

**Fig. 13:**
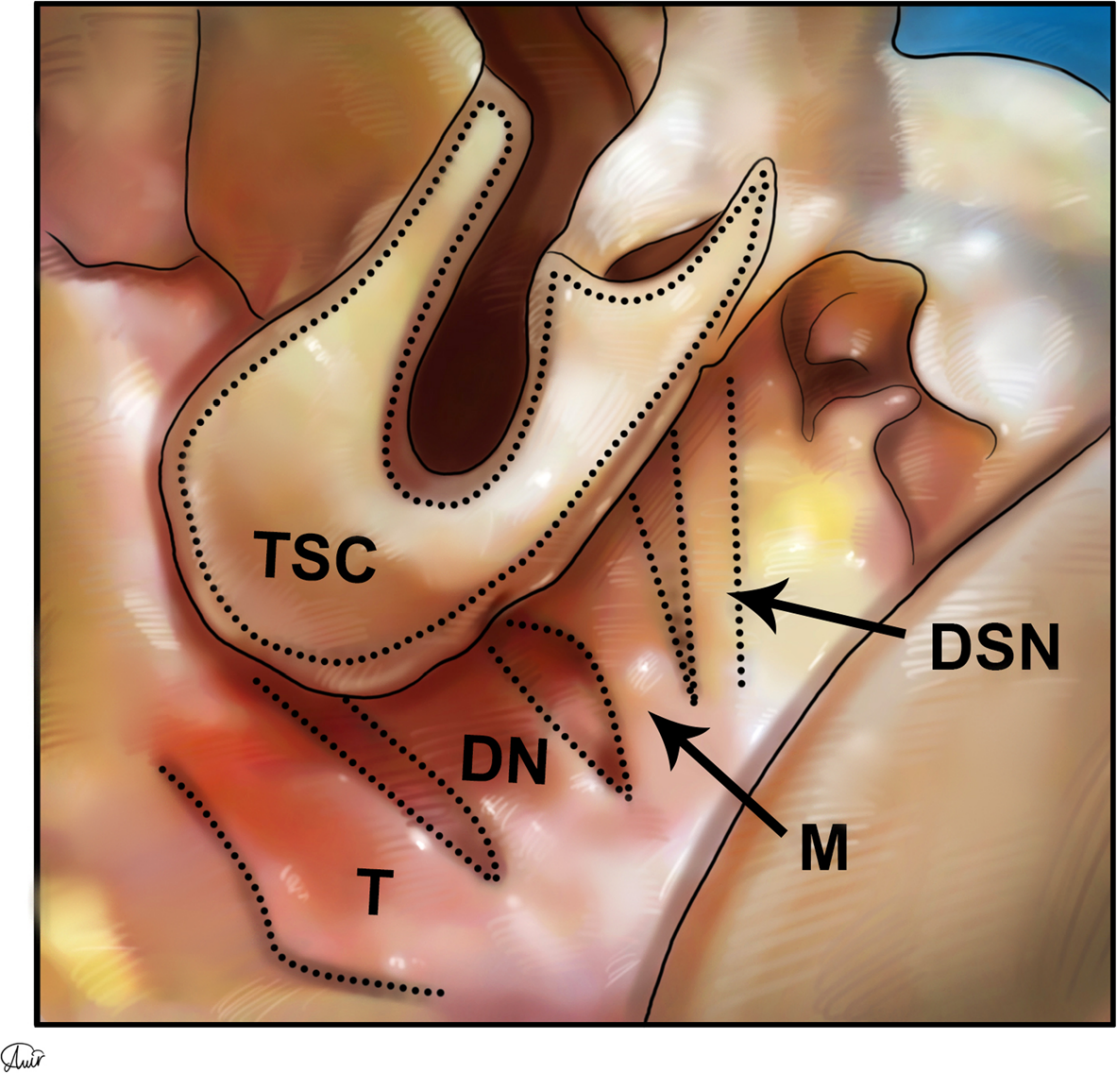
Tela subcutanea cutis (TSC), dilator naris (DN), mytriformis (M), and depressor septi nasalis (DSN) muscles can be seen in this schematic figure of the nasal area

**Fig. 14:**
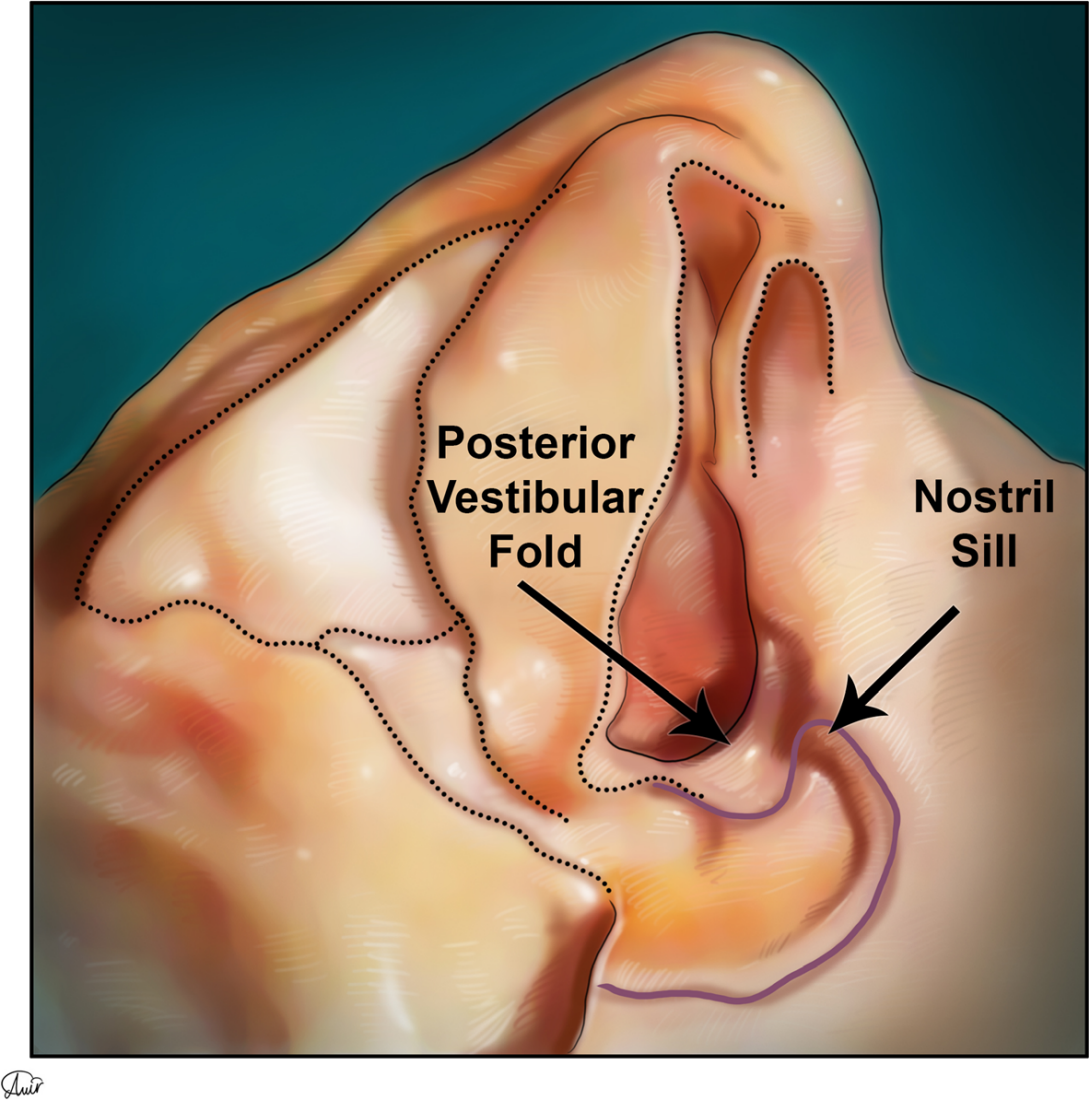
The posterior vestibular fold, located adjacent to the nostril sill, is where the alveolar process separates from the nasal chamber

Muscle insertions of the nostril sill area include the depressor septi nasalis, myrtiformis, and dilator naris (DN) muscles, which originate from the maxilla and insert into the soft tissue and skin of the nares. The tela subcutanea cutis (TSC) that can be seen in this area (Figs. 12, 13, and 14) is a folded layer of dermis and subcutaneous tissue that connects the lateral and medial crura when seen from the basal view.

**Fig. 15:**
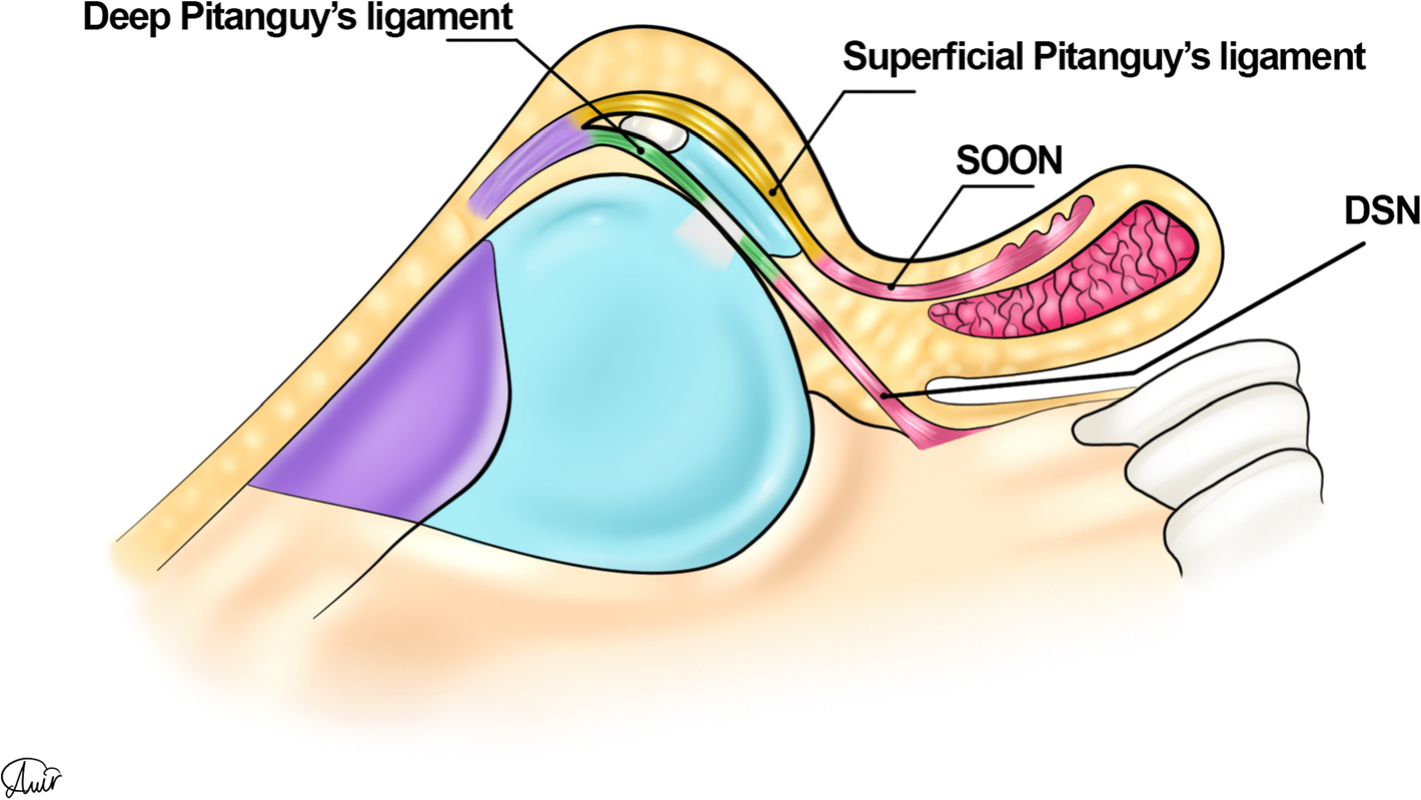
Superficial and deep pitanguy ligaments continue caudally as the superficial orbicularis oris nasalis (SOON) and depressor septi nasalis (DSN) muscles respectively

Other anatomical considerations in the sill area include the superficial and deep pitanguy’s ligaments, which extend caudally between the lower lateral cartilages and continue along the superficial orbicularis oris nasalis (SOON) and depressor septi nasalis (DSN) muscles, respectively (Fig. 15).

Our experience in the present study revealed that the proper symmetry and shape of the alar base and nostrils are dependent on the precise evaluation and further reconstruction of the nasal sill dimensions, especially in unilateral deformities where the normal shape of the sill is achieved similar to the normal side. In minor sill defects, muscle repositioning, specific suturing techniques, and small soft tissue grafts may result in the satisfactory elevation of the sill area [19]; however, in larger defects, composite grafts may be required to achieve the desired clinical outcomes [27].

Among esthetic rhinoplasty patients, those who require nasal tip modification and correction of gross septal deviation or perforation, as well as those who undergo esthetic rhinoplasty through an open approach, augmentation of the sill area can be performed using an open approach if needed (Fig. 1b). Also, in esthetic rhinoplasty patients, who require alar base reduction and have defects in the sill area, insertion of the sill graft through the alar base incision can be highly useful (Fig. 1c).

The review of published literature, addressing the concept of nasal sill augmentation, revealed that cleft palate patients require major corrections for sill defects (Table 2). Therefore, special attention must be paid to nasal sill reconstruction in these patients. However, nasal sill reconstruction in these patients is not usually performed as an independent procedure but as part of the cleft repair process. Dissection and repositioning of the orbicularis oris and depressor septi muscles is often the most preferred technique for sill augmentation in these patients [13, 23, 24]. Repositioning of the medial and lateral flaps of the upper lip during cleft closure is another method for reconstruction of the sill area [22]. Although no major complications were reported for this method, the absence of graft can occasionally result in further depression of the sill area in the long term.

As mentioned earlier, no complications were detected among the participants of our study; however, infection, bleeding, ischemia, flap necrosis, complications associated with the harvesting procedure, graft deviation, obvious scar, excessive decrease in the nostril size, impaired ventilation, shortening of the upper lip, and sensory dysfunction are among potential complications, which require strict considerations, especially in the follow-up examinations [10, 20, 23, 25].

Earlobe-derived cartilage grafts do not offer satisfactory esthetic results in the sill area and are associated with complications in some cases [20]. Instead, alveolar bone grafting in 18 unilateral cleft lip and palate patients with tension-free sutures produced optimal esthetic outcomes in the nasal sill area [19], with significant improvement in the width and height measures of the cleft site.

A review of previous studies showed that many of the published techniques are based on the transposition of flaps [13, 14, 18, 22–24]. The application of these techniques may be justified for patients with clefts or those with malignancies, where a part of the soft tissue is usually deficient. However, the use of these techniques in patients, who are diagnosed with simple congenital defects, seems extremely aggressive. On the other hand, the conservative design of our technique and the lack of extensive flaps provide an opportunity for nasal sill reconstruction in patients with congenital defects. In other words, the non-invasive design of our technique and providing a solution for nasal sill reconstruction in patients with congenital defects can be considered the most significant advantages of this study. However, the possibility of using our technique for patients with clefts, malignancies, or traumatic lesions cannot be rejected.

Recurrent asymmetry following graft deformity may be the most important limitation of this technique. Overall, ensuring that the graft is stable and fixed in its position can be very helpful in preventing the occurrence of this complication. However, in such cases, a secondary revision intervention is required.

In general, one of the advantages of this procedure is that it is not technique-sensitive, and it is easy to perform. Also, this technique is repeatable and does not produce a remarkable scar in the surgical site. On the other hand, its disadvantage is donor site morbidity.

In conclusion, based on the findings of the present study, our novel technique can be successfully used for reconstructing the nasal sill area, with minimal complications and morbidities in patients, who require esthetic rhinoplasty or have congenital defects, cleft lip deformities, malignancies, or traumatic lesions. It should be noted that in this technique, the proper symmetry and shape of the alar base and nostrils are dependent on the precise evaluation and further reconstruction of the nasal sill dimensions.

## Data Availability

All of the data of the patients are available and could be sent to the reviewers, if it is needed.

## Abbreviations

ROF: Rhinoplasty Outcome Evaluation Form
DN: Dilator naris
SOON: Superficial orbicularis oris nasalis
DSN: Depressor septi nasalis
TSC: Tela subcutanea cutis
